# Relative validity of a food frequency questionnaire for adolescents from a capital in the Northeastern region of Brazil

**DOI:** 10.1590/1414-431X20209991

**Published:** 2020-12-21

**Authors:** E.G. Bogea, A.K.T.C. França, M.L.B.M. Bragança, J.S. Vaz, M.C. Assunção, M.A. Barbieri, H. Bettiol, A.A.M. Silva

**Affiliations:** 1 Centro de Ciências Biológicas e da Saúde, Programa de Pós-Graduação em Saúde Coletiva, Universidade Federal do Maranhão, São Luís, MA, Brasil; 2 Faculdade de Nutrição, Programa de Pós-Graduação em Nutrição e Alimentos, Universidade Federal de Pelotas, Pelotas, RS, Brasil; 3 Programa de Pós-Graduação em Epidemiologia, Universidade Federal de Pelotas, Pelotas, RS, Brasil; 4 Programa de Pós-Graduação em Saúde Pública, Universidade Federal do Rio Grande, Rio Grande, RS, Brasil; 5 Departamento de Puericultura e Pediatria, Faculdade de Medicina de Ribeirão Preto, Universidade de São Paulo, Ribeirão Preto, SP, Brasil

**Keywords:** Validation studies, Food consumption, Adolescent, Cohort studies

## Abstract

The present study was conducted to evaluate the validity of the Food Frequency Questionnaire (FFQ) used in the RPS Birth Cohort Consortium (Ribeirão Preto, Pelotas, and São Luís) to assess dietary intake of adolescents from São Luís, Maranhão. The research was developed with 152 adolescents aged 18 and 19 years. For the validation of the FFQ, the average of three 24-hour recalls (24HRs) was used as the reference method. The mean and standard deviation of energy and nutrient intake extracted from the surveys were estimated. The paired Student's *t*-test was used to verify the differences between the instruments. Pearson correlation coefficient, intraclass correlation coefficient (ICC), weighted Kappa, and the Bland-Altman plot were calculated in order to measure the agreement. The study adopted a level of significance <5%. Compared with the three 24HRs, the FFQ overestimated the consumption of most nutrients. Energy-adjusted and de-attenuated concordance Pearson correlation coefficients ranged from 0.06 to 0.43, and correlations were significant for iron, calcium, riboflavin, sodium, saturated fat, niacin, and vitamin C. The energy-adjusted and de-attenuated ICCs ranged from 0.01 to 0.31, and the weighted Kappa ranged from 0.01 to 0.46. The analyses of agreement were significant for vitamin C, fiber, calcium, riboflavin, niacin, sodium, lipids, and iron. In conclusion, the FFQ presented acceptable relative validity for lipids, saturated fatty acids, fiber, calcium, iron, riboflavin, niacin, vitamin C, and sodium. This instrument will be useful in studies about food consumption of adolescents in São Luís, Maranhão.

## Introduction

Adolescents constitute a nutritionally vulnerable group due to their elevated nutritional demands, consumption of junk food, and susceptibility to environmental influences (1). Information about food habits, nutrition, and monitoring of dietary intake are important to identify risk behaviors, assure the full potential of the development of adolescents, and enable intervention to prevent more harm in the adult phase (2,3).

A reliable assessment of food consumption demands appropriate measurement instruments with great accuracy and high reliability (4). Thus, the selection of the dietary method must be guided by the objective of the investigation and by the characteristics of the target population.

Among multiple dietary intake measures, the Food Frequency Questionnaire (FFQ) stands out for being an easy-to-use and cost-effective tool, able to evaluate the habitual dietary intake of population groups and a high number of food items. In contrast, the FFQ presents certain limitations, such as dependency on the memory of the interviewees about past food habits, low accuracy in quantifying food consumption due to the use of standardized measurements, and loss of details of food consumption (4,5).

The developed FFQs must be validated due to inaccuracy and differences among the populations of interest, presenting errors that could be related to individuals, to the instrument itself, or to external effects (6). The term validity is generally defined as a degree to which an instrument measures what it is supposed to measure. Instrument validation is considered a crucial process since errors in measurement reduce the estimates achieved through epidemiological studies (7). The validation of a FFQ is determined by an evaluation of instrument performance when its estimation of food and nutrient consumption is compared to the measurements obtained through other independent methods (8,9).

Despite the existence of some validated FFQs for Brazilian adolescents, most of them did not assess specifically this stage of life or were performed in the Southern and Southeastern regions of the country, failing to represent vast social and cultural variations in the Northeastern region. The study conducted by Araujo et al. (10) in Rio de Janeiro with 169 adolescents evaluated the relative validity of a semi-quantitative FFQ using 24-h food records or recalls (24HRs) as the reference method. The authors observed weak to moderate correlation coefficients, with statistical significance for all of the evaluated nutrients. The FFQ was considered an appropriate instrument to classify the energy and nutrient consumption in the studied group.

Given the importance of reliable knowledge of adolescents' food consumption (11), it is essential to validate instruments of food consumption evaluation in different regions of the country. Thus, the present study aimed to validate the FFQ used in the RPS study in a capital from the Northeastern region of Brazil.

## Material and Methods

### Study design

This is a cross-sectional study that used data from a Brazilian cohort research named “Determinants throughout the life cycle of obesity, precursors of chronic diseases, human capital, and mental health-RPS Birth Cohort Consortium (Ribeirão Preto, Pelotas and São Luís)”, developed in the cities of São Luís (MA; Northeastern region), Ribeirão Preto (SP; Southeastern region), and Pelotas (RS; Southern region), Brazil. The study used information from the cohort in São Luís, which was approved by the Research Ethics Committee from the Hospital of Universidade Federal do Maranhão (process No. 1.302.489). All of the participants signed the free and informed consent form.

### Population and sample of the study

The perinatal study of the São Luís cohort was started at birth in ten public and private hospitals in the city from March 1997 to February 1998. The researchers used systematic sampling with proportional stratification according to the number of births in each maternity unit (1 of every 7 births). The sample tended to represent the births in the city during that period, considering that the births in hospitals represented 96.3% of the total. Cases of multiple births, stillbirths, and refusal or impossibility to locate the mother were excluded and represented 5.8% of the total, resulting in a sample of 2,443 births in hospitals.

The cohort in São Luís was assessed again at 7 to 9 years of age (2005 to 2006) through a complex sampling design, using the birth weight variable to define the sample that was necessary for the school age assessment. The participation rate was 72.7% (673 participants). Details of methods were published elsewhere (12).

In 2015, the participants were invited to return for a new evaluation at 18 years of age. Two strategies were developed to locate the adolescents: a search in the military enlistment records (only for boys) and schools (for both boys and girls). In 2016, the participants were scheduled to undergo the evaluation and the sample was restricted to the adolescents that participated in the birth cohort (687 adolescents). To increase the power of the sample and prevent future losses, the cohort was opened to include other individuals born in São Luís in the year of 1997. The first stage of the search occurred using the SINASC system (Information System on Live Birth). From this list, a random selection was made obtaining a total of 4,593 adolescents born in 1997. Of these, it was possible to make telephone or personal contact with 1,716 adolescents. In a second stage, volunteers born in the same year were identified in schools, universities, and social media, totaling 1,831 adolescents.

### Procedures for data collection

After identifying the participants of the current follow-up, the adolescents were contacted to schedule the cohort assessment that consisted of a general questionnaire, anthropometric measures, and blood collection. All interviews and assessments were conducted by trained research assistants.

The general questionnaire was a standardized interview applied by the RPS cohort consortium composed by six sections (A-F). The following data were used in the current study: sex (female and male), age in years (mean and standard deviation), social class (A, B1 and B2, C1 and C2, D, and E), education level (elementary school, high school, and higher education/degree programs), skin color (white, brown, black, and “yellow”), and nutritional status (underweight, normal weight, overweight, and obese). The nutritional status was classified according to WHO standards (13) using the z-score of body mass index [BMI (kg/m^2^) for age and sex, and classified using the following cut-offs (BMI for age): underweight (≤-1 SD), normal weight (>-1 SD and <+1 SD), overweight (≥+1 SD and ≤+2 SD), and obese (>+2 SD)].

### Relative validation study

All 2,516 adolescents in the São Luis RPS cohort answered the FFQ. A subsample of 200 participants was randomly selected for the validation study taking into consideration the minimum and maximum sizes of the sample to perform the validation of the FFQ (7,11).

The 24HR was the dietary instrument selected as the reference method, which is widely used in studies of FFQ validation (8,10). Three 24HRs were administered, from which two referred to weekdays (from Monday to Friday) and one referred to weekends/holidays.

According to Carroll et al. (14), it is possible to obtain adequate correlation coefficients for a validation study with two to five repeated measurements. The three 24HRs were administered during a period of 30 to 60 days after answering the FFQ, with a minimum interval of 15 days between each recall. Two 24HRs were answered in-person and one by telephone.

**Figure 1:**
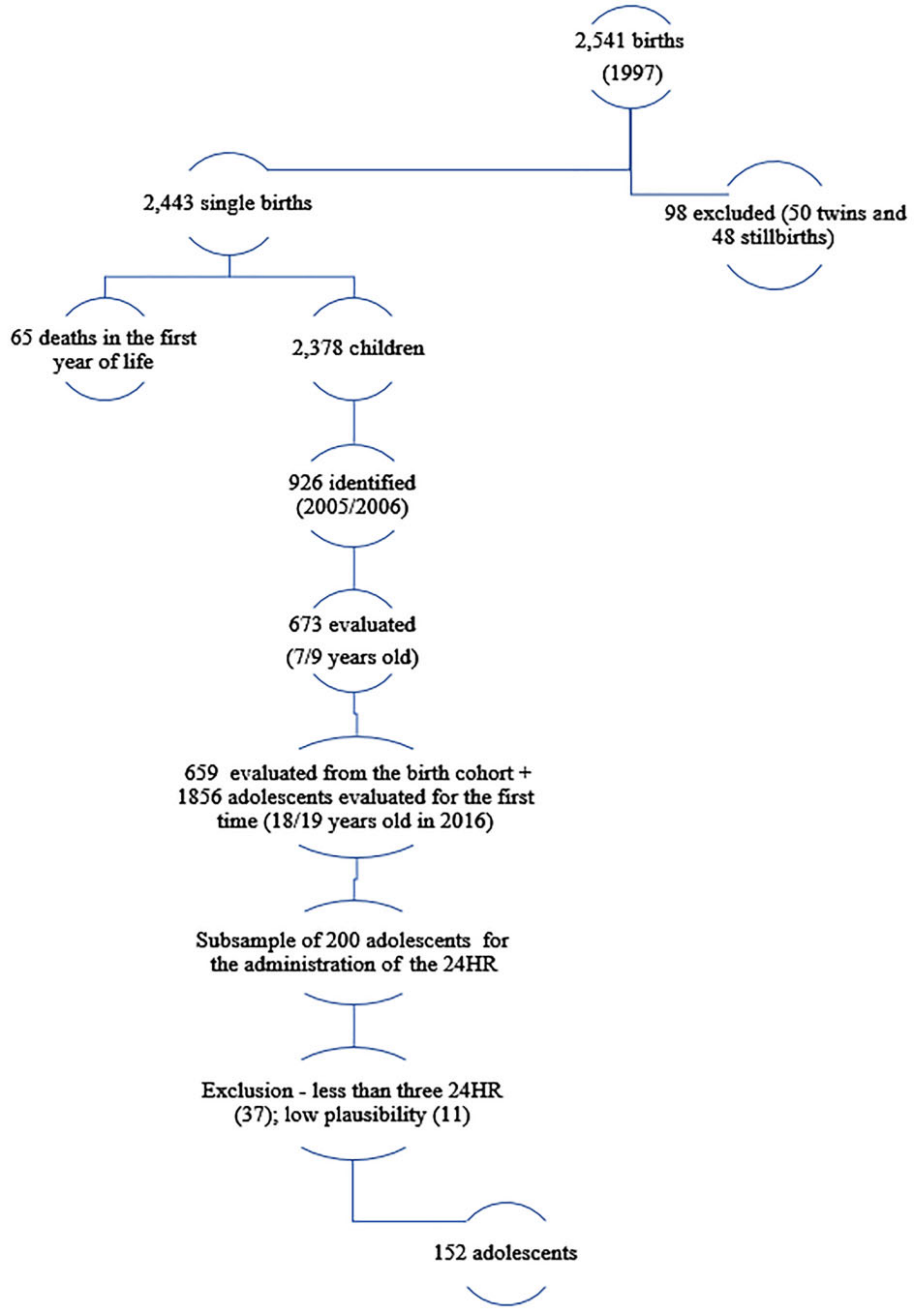
Sampling of the São Luís, MA cohort 1997-98/2005-06/2016 and the adolescents evaluated for the first time (2016). 24HR: 24-h recall.

After completing the protocol, we excluded 37 subjects who did not complete the three 24HRs, and another 11 subjects who reported implausible energy reports. The plausibility was determined by a comparison between the reported energy intake and the estimated energy requirements (15,16). The energy intake was considered implausible when the ratio between the reported energy intake and the energy requirement was out of the range of ±2 standard deviation. Thus, 254 subjects were considered for the validation study (Figure 1).

### Food frequency questionnaire

The FFQ used in the present study was a semiquantitative questionnaire regarding habitual consumption in the past 12 months. This instrument was developed by Schneider et al. (4) and adapted by the nutritionists involved in the São Luis birth cohort. The main changes in the original FFQ were related to specific food portion size, exclusion of regional foods of the Southern region (fried polenta/cassava, “chimarrão”, cod, strawberry, and peas), and inclusion of regional foods from of Northeastern region (tapioca/couscous, jussara, chard, vinegar, cabbage, gherkin, okra, crab, stuffed cake, guarana powder, energy drinks). Additionally, another six food items were included considering current changes in the food market (e.g., sushi, sashimi, breakfast cereals, cereal bars, nuggets, and instant noodles). The questionnaire was administered by nutritionists properly trained with the assistance of REDCap, a web application that is safe for the creation and management of online research and databases (17).

This instrument was composed of 89 food items divided into 7 food groups: cereals and tubers; milk and dairy products; fruits and vegetables; meat and eggs; candies; drinks; other foods. The participants were asked about the frequency and the quantity of consumption of each food item. Eight options of answers were used for the frequency of consumption: never or less than once/month; 1 to 3 times/month; 1 to 2 times/week; 3 to 4 times/week; 5 to 6 times/week; once/day; 2 to 4 times/day; more than 5 times/day. To estimate the amount of food intake, an average portion size was presented for each item, and the participant informed if the usual portion consumed was similar, bigger, or smaller than the reference portion size. The reference of portion size adopted was defined by the administration of two 24HRs in a sub-study of 185 adolescents conducted in 2006 (18). The portion size of regional food items was established in an agreement among nutritionists involved in the current project, taking into consideration what is commonly consumed by the adolescent population in São Luís. Data from the Brazilian Household Budget Survey (POF, acronym in Portuguese) were also used to determine the portion size of specific food items (19).

### 24-h recall

First, participants were asked to describe all foods and beverages consumed during the previous day from their wake-up time until their last meal, without interruptions by the interviewer. Subsequently, the interviewer requested a detailed description of each food and beverage reported, meal time, preparation, food brand, and portion sizes. Finally, a review of all reported items was made (20). To reduce recall biases and to assist in the identification of the estimated portion size, the interviewers used a photo album with pictures of the home utensils and food portions as support material (21).

The software STATA 14.0 (<https://www.stata.com/stata14/>; StataCorp, USA) was used to analyze the food consumption data from the 24HR and the FFQ. Portion size was converted into grams or milliliters using a standard reference table (22).

The energy and nutrient intake were estimated using the Brazilian Food Composition Table (23), complemented with the United States Department of Agriculture (USDA) National Nutrient Database for Standard Reference (24).

### Statistical analysis

Participants are described according to sociodemographic and nutritional status. Categorical variables are described by frequencies and continuous variables by mean and standard deviation.

The following nutrients were selected for analyses: energy, proteins, carbohydrates, fiber, calcium, iron, thiamine, riboflavin, niacin, vitamin C, sodium, cholesterol, saturated, monounsaturated, and polyunsaturated fatty acids. Data were transformed (log_10_) to optimize distribution normality. Nutrient intakes were energy-adjusted using Willet's residual method (7). In order to compare the average consumption of nutrients between the FFQ and the average of the three 24HRs, dependent samples tests were used. The paired Student's *t*-test was applied with logarithmic transformations of the variables.

In order to correct for within-individual error in the measurement of the average of the three 24HRs, which tends to reduce correlation coefficients toward zero, the correlation coefficient found was multiplied by the de-attenuation factor (1+(σ^2^_w_/σ^2^_b_)/n)^0.5^, where σ^2^_w_ is the within-individual variance, σ^2^_b_ is the between-individual variance, and n represents the number of replicate measurements (n=3) (7). The within- and between-individual variance components were determined by a random-effects model with recorded intake as the dependent variable and subject identification number as the independent variable (25). De-attenuated correlations represent values after between- and within-individual variance correction.

To test the FFQ against the average of the three 24HRs, multiple correlation and agreement methods were used. Crude, energy-adjusted, and de-attenuated Pearson correlation coefficients were calculated between energy and nutrients estimated from the FFQ and the average of the three 24hRs. The following cutoffs were applied to interpret Pearson correlation coefficients: r=0.10 to 0.30 (weak); r=0.40 to 0.60 (moderate); r=0.70 to 1.00 (strong) (7,26).

The intraclass correlation coefficients (ICC) of log-transformed crude, energy-adjusted, and de-attenuated nutrients were calculated in order to evaluate the reliability and homogeneity between methods. ICC above 0.40 are considered a good agreement between the methods (27).

The agreement of energy-adjusted nutrient intake, the FFQ, and the three 24HRs was performed using weighted Kappa statistics. The following Kappa interpretation was applied: 0.0-0.20 (slight), 0.21-0.40 (fair), 0.41-0.60 (moderate), 0.61-0.80 (substantial), 0.81-1.0 (almost perfect) (28).

Bland and Altman graphs (29) were plotted to evaluate the limits of agreement and the magnitude of the differences between the mean of energy and nutrient estimates in both methods. All analyses adopted the level of significance of <5%.

## Results

Of the 152 participants, 63.7% were female, 64.2% reported brown skin color. Fifty-five percent had completed high school, 94.1% were single, and 48% were from the C socioeconomic class. The average age was 18.2±0.4 years. The average BMI was 21.8 kg/m^2^, with 17.8% being overweight.

The estimated energy and nutrient intakes by the FFQ were greater than the three 24HRs for most nutrients, except for niacin and mono- and polyunsaturated fatty acids. The lowest differences between methods were observed for protein, sodium, and monounsaturated fat consumption. No significant difference for protein, sodium, and mono- and polyunsaturated fatty acids estimations were observed (Table 1).

**Table 1:** Estimated nutrient intake with the 24-hour recalls (24HRs) and the food frequency questionnaire (FFQ).

Nutrients	Estimate of daily nutrient intake				P-value
	FFQ		24HR		
	Mean	SD	Mean	SD	
Carbohydrate (g/day)	418.56	38.77	303.81	36.90	<0.001
Protein (g/day)	95.47	17.10	93.40	22.81	0.133^*^
Lipids (g/day)	69.26	13.87	61.68	2.14	<0.001
Saturated fatty acids (g/day)	26.09	6.03	22.94	5.40	<0.001
Monounsaturated fatty acids (g/day)	20.56	4.83	20.64	4.97	0.959^*^
Polyunsaturated fatty acids (g/day)	11.99	3.42	12.61	4.42	0.540^*^
Fiber (g/day)	73.41	23.43	20.61	6.31	<0.001
Calcium (mg/day)	729.09	253.74	372.62	150.21	<0.001
Iron (mg/day)	12.54	3.23	11.33	2.81	<0.001
Thiamine (mg/day)	1.428	0.34	1.14	0.32	<0.001
Riboflavin (mg/day)	1.827	0.51	1.50	0.54	<0.001
Niacin (mg/day)	14.83	4.35	18.77	7.42	<0.001
Vitamin C (mg/day)	134.98	87.85	84.13	58.28	<0.001
Sodium (mg/day)	1,825.03	479.00	1,810.18	506.11	0.577^*^
Cholesterol (mg/day)	384.23	145.31	347.61	347.61	0.008

*P>0.05, Student's *t*-test for the difference of mean after logarithmic transformation.

Crude Pearson correlation coefficients were significant for carbohydrates, lipids, fiber, calcium, thiamine, riboflavin, vitamin C, and sodium, ranging from 0.09 (cholesterol) to 0.42 (vitamin C). After the adjustment for energy intake and the de-attenuation, there was a reduction in the coefficient for most nutrients, except calcium, iron, niacin, and vitamin C, whose coefficients increased, and fiber, which kept the same coefficient. The adjusted and de-attenuated coefficients were significant for nutrients such as iron, calcium, riboflavin, sodium, saturated fat, niacin, and vitamin C, ranging from 0.06 (cholesterol) to 0.43 (vitamin C). Vitamin C stood out as the only nutrient with a coefficient greater than 0.40 (Table 2).

**Table 2:** Correlation between nutrient intake measured with the 24-hour recalls and the food frequency questionnaire.

Nutrients	Pearson correlation coefficient (r)		
	Crude^*^	Energy-adjusted^*^	Energy-adjusted and de-attenuated^*^
Carbohydrate (g/day)	0.34^¥^	0.07	0.07
Protein (g/day)	0.11	0.11	0.11
Lipids (g/day)	0.22^†^	0.11	0.12
Saturated fatty acids (g/day)	0.20	0.18^†^	0.19
Monounsaturated fatty acids (g/day)	0.18	0.14	0.15
Polyunsaturated fatty acids (g/day)	0.20	0.10	0.10
Fiber (g/day)	0.28^†^	0.28	0.31
Calcium (mg/day)	0.20^¥^	0.30^¥^	0.33
Iron (mg/day)	0.20	0.21^†^	0.22
Thiamine (mg/day)	0.24^†^	0.10	0.10
Riboflavin (mg/day)	0.26^†^	0.20^†^	0.20
Niacin (mg/day)	0.25	0.29^¥^	0.30
Vitamin C (mg/day)	0.42^¥^	0.41^¥^	0.43
Sodium (mg/day)	0.30^†^	0.22^†^	0.24
Cholesterol (mg/day)	0.09	0.06	0.06

*Coefficients of the nutrients after logarithmic transformation. ^†^P<0.05; ^¥^P<0.001.

**Figure 2:**
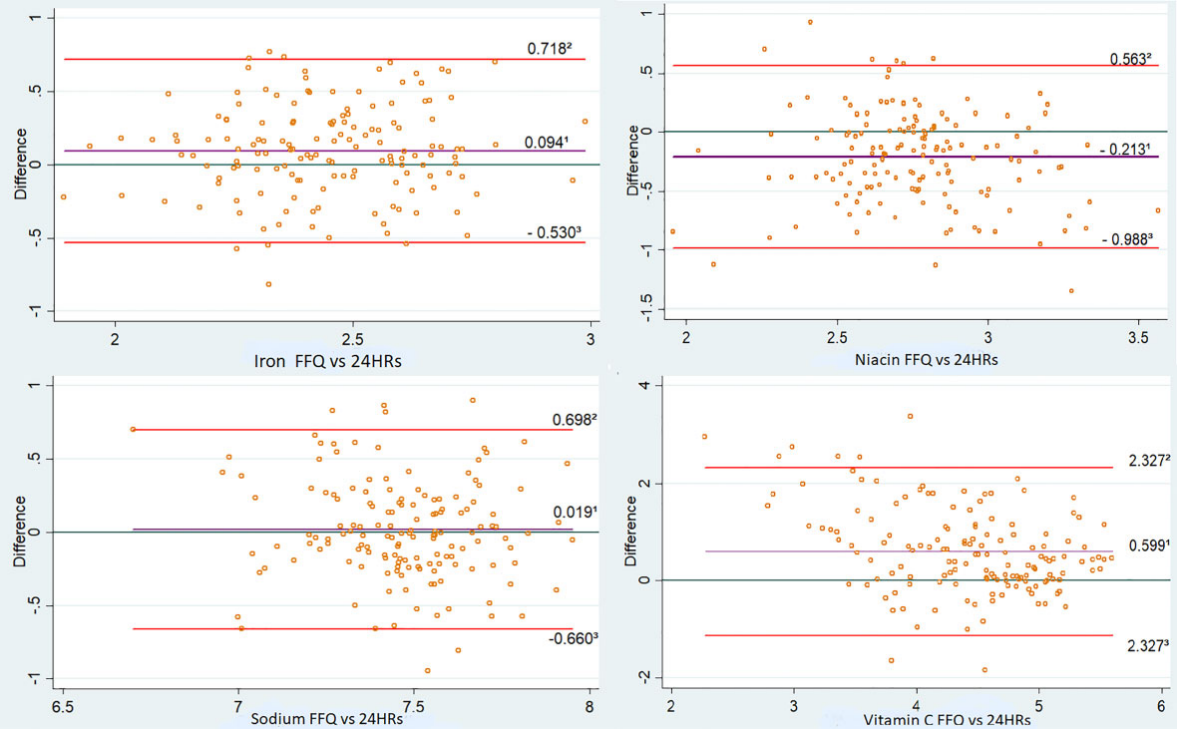
Mean difference of the three 24-hour recalls (24HRs) and the food frequency questionnaire (FFQ) and superior and inferior limits of agreement with log-transformed data for iron, niacin, sodium, and vitamin C. São Luís, Maranhão, RPS Cohort Consortium 2016 (n=152).

The adjusted and de-attenuated ICCs ranged from 0.01 (carbohydrate) to 0.31 (vitamin C). Although the coefficients showed low agreement between methods, such agreement was significant for saturated fatty acids, fiber, calcium, iron, riboflavin, niacin, vitamin C, and sodium (Table 3). Through the Bland-Altman plots, it is possible to visualize the agreement and the magnitude of the differences between the FFQ and the average of the three 24HRs (Figure 2).

**Table 3:** Interclass correlation coefficient of nutrient intake measured with the 24-hour recalls and the food frequency questionnaire.

Nutrients	Intraclass correlation coefficient^*^ (95%CI)		
	Crude	Energy-adjusted	Energy-adjusted and de-attenuated
Carbohydrate (g/day)	0.36 (0.12; 0.32)^†^	0.01 (-0.02; 0.04)	0.01
Protein (g/day)	0.11 (-0.04; 0.26)	0.10 (-0.05; 0.25)	0.10
Lipids (g/day)	0.21 (0.06; 0.34)^†^	0.10 (-0.04; 0.23)	0.10
Saturated fatty acids (g/day)	0.18 (0.04; 0.32)^†^	0.15 (0.02; 0.29)^†^	0.17
Monounsaturated fatty acids (g/day)	0.17 (0.02; 0.32)^†^	0.14 (-0.02; 0.30)	0.15
Polyunsaturated fatty acids (g/day)	0.20 (0.04; 0.35)^†^	0.09 (-0.06; 0.24)	0.10
Fiber (g/day)	0.15 (0.07; 0.24)^†^	0.03 (0.01; 0.04)^†^	0.03
Calcium (mg/day)	0.15 (0.07; 0.22)^†^	0.11 (0.05; 0.17)^†^	0.12
Iron (mg/day)	0.18 (0.04; 0.32)^†^	0.20 (0.05; 0.34)^†^	0.21
Thiamine (mg/day)	0.20 (0.07; 0.33)^†^	0.07 (-0.04; 0.18)	0.07
Riboflavin (mg/day)	0.22 (0.09; 0.35)^†^	0.16 (0.03; 0.29)^†^	0.16
Niacin (mg/day)	0.23 (0.09; 0.37)^†^	0.24 (0.11; 0.36)^†^	0.25
Vitamin C (mg/day)	0.32 (0.21; 0.43)^†^	0.30 (0.19; 0.41)^†^	0.31
Sodium (mg/day)	0.29 (0.15; 0.44)^†^	0.21 (0.06; 0.36)^†^	0.23
Cholesterol (mg/day)	0.09 (-0.06; 0.24)	0.06 (-0.09; 0.21)	0.06

*Coefficients of the nutrients after logarithmic transformation. ^†^P<0.05.

The weighted Kappa of energy-adjusted nutrients ranged from 0.01 for carbohydrate to 0.46 for vitamin C. The agreement between the methods was significant for lipids, fiber, calcium, iron, riboflavin, niacin, vitamin C, and sodium. There was moderate agreement for vitamin C, fair agreement for fiber, calcium, riboflavin, niacin, and sodium, and slight agreement for lipids and iron (Table 4).

**Table 4:** Crude and adjusted weighted kappa of nutrient intake measured with the 24-hour recalls and the food frequency questionnaire.

Nutrients	Weighted Kappa^*^ (95%CI)	
	Crude	Energy-adjusted
Carbohydrate (g/day)	0.23 (0.08−0.36)^†^	0.01 (−0.16−0.16)
Protein (g/day)	0.07 (−0.07−0.24)	0.07 (−0.07−0.22)
Lipids (g/day)	0.22 (0.06−0.37)^†^	0.17 (0.01−0.30)^†^
Saturated fatty acids (g/day)	0.19 (0.03−0.34)^†^	0.14 (−0.03−0.27)
Monounsaturated fatty acids (g/day)	0.12 (−0.05−0.29)	0.12 (−0.06−0.26)
Polyunsaturated fatty acids (g/day)	0.15 (0.01−0.32)^†^	0.13 (−0.05−0.29)
Fiber (g/day)	0.22 (0.08−0.40)^†^	0.23 (0.08−0.41)^†^
Calcium (mg/day)	0.34 (0.18−0.47)^†^	0.27 (0.12−0.42)^†^
Iron (mg/day)	0.15 (−0.01−0.30)	0.19 (0.01−0.32)^†^
Thiamine (mg/day)	0.17 (0.02−0.32)^†^	0.10 (−0.06−0.26)
Riboflavin (mg/day)	0.22 (0.05−0.37)^†^	0.20 (0.03−0.34)^†^
Niacin (mg/day)	0.27 (0.13−0.42)^†^	0.22 (0.04−0.37)^†^
Vitamin C (mg/day)	0.40 (0.25−0.55)^†^	0.46 (0.32−0.59)^†^
Sodium (mg/day)	0.25 (0.08−0.38)^†^	0.21 (0.07−0.37)^†^
Cholesterol (mg/day)	0.11 (−0.05−0.26)^†^	0.07 (−0.01−0.25)

*Coefficients corresponding to the quartile of nutrient consumption. ^†^P<0.05.

## Discussion

The present study analyzed the relative validity of a digital semiquantitative FFQ with 89 items developed to evaluate the food habits of adolescents from São Luis, a capital in the Brazilian Northeastern region. The average of three 24HRs was used as the reference method. The comparison between nutrients estimated from the FFQ and the three 24HRs indicated an overestimation of the FFQ for most nutrients, but no significant difference in the consumption of protein, sodium, and mono- and polyunsaturated fatty acids were observed. The energy-adjusted correlation coefficients were significant for iron, calcium, riboflavin, sodium, saturated fat, niacin, and vitamin C - the latter presenting a moderate correlation. The agreement analyses were significant for vitamin C, fiber, calcium, riboflavin, niacin, sodium, lipids, and iron.

Some aspects can be pointed out as limitations of the study, since there is no reference method for evaluation of food consumption that is considered to be the gold standard. Although the 24HR is vastly used in validation studies, this method presents sources of errors that are similar to the FFQ, considering that both methods require the use of memory by the interviewee (7). Another limitation is that the FFQ used was adapted from a FFQ developed for adolescents and adults from Pelotas, Southern Brazil (4). Adaptations in portion sizes were made in order to minimize this limitation, together with the inclusion of regional foods and the exclusion of foods that are not part of the local dietary habits.

The strengths of this study are the satisfactory sample size for validation studies, the methodology used for validation, and appropriate statistical procedures for evaluation of agreement between methods, including weighted Kappa, ICC, Bland-Altman, and Pearson's correlation agreement statistical analyses.

The validation process of a FFQ is a fundamental step for the evaluation of food habits. In this context, the choice of an appropriate reference method is crucial. However, the main limitation in FFQ validations is the lack of a gold standard method (30). In the present study, the 24HR was used as reference method due to its high acceptance and capability of measuring with high details the portion sizes and variety of the food consumed. Studies indicate that the 24HR is the most appropriate reference method for epidemiological studies regarding validation of food consumption, with 75% of the validation studies using it for comparison with the FFQ (31,32).

The overestimation of the FFQ in relation to the 24HR occurred for most nutrients, except niacin, iron, and mono- and polyunsaturated fatty acids. This finding is similar to those of other studies with adolescents (8,16,33). The under- and overestimation of the FFQ observed in the current study can be attributed to the characteristics of the instrument, such as the long list of foods (34). A FFQ containing 89 foods is considered long and may induce the error due to an extensive number of questions. Despite this, the FFQ was able to provide a valid measure for specific nutrients.

Weak correlation coefficients were observed for most nutrients, except for vitamin C indicating a moderate correlation according to the weighted Kappa. In all of the analyses, there was a reduction in the coefficients after the adjustment for energy for most nutrients, except iron, niacin, and vitamin C - the latter only in the weighted Kappa. Adjustment for energy is performed on the premise that each individual describes the nutrient intake similarly in both methods. The correlation coefficients increase when the variability of the estimates for nutrient consumption is associated to the energy intake and reduce when the variability is related to the under- or overestimation of food consumption (7,34). In the present study, a reduction in the coefficients was more frequent, justified by the overestimation of the FFQ compared to the 24HR. A tendency in coefficient reduction after the adjustment for energy was also observed in studies by Crispim et al. (34) e Zanolla et al. (35).

The adjusted and de-attenuated Pearson correlation coefficient was higher for vitamin C (0.43). Significant result was also observed for nutrients such as iron, calcium, riboflavin, sodium, saturated fat, and niacin, but with low correlation. Correlation coefficients higher than 0.7 are rarely reported in validation studies of dietary methods due to the complexity of human diet and the inexistence of a gold standard reference method (36). Additionally, the correlation coefficients are significantly higher when the reference method is administered for 8 to 14 days (9).

A study conducted by Araujo et al. (10) with adolescents from the state of Rio de Janeiro (Brazil) also used the average of three 24HRs as the reference method and observed similar results to ours with Pearson correlation coefficients ranging from 0.33 to 0.46, and most of the nutrients presenting low correlation. However, the studies conducted by Henn et al. (33), Martinez et al. (31), and Mascarenhas et al. (8) with Brazilian adolescents observed higher coefficients, but greater correlation for a few nutrients. Despite the vast use of Pearson correlation in FFQ validation studies, such analysis evaluates only the linear association between the variables, not the agreement between the methods (37,38).

Regarding the ICC, there was a variation of the coefficients (0.01 to 0.31) lower than 0.40 for all of the nutrients indicating low correlation. The agreement analyses, such as the ICC, evaluate the coincidence between values. For this reason, results are usually lower than the correlation coefficient when applied to the same dataset (37,38). There are very few studies that use the ICC in the analysis of FFQ validation for adolescents in Brazil. Martinez et al. reported ICCs ranging from 0.02 to 0.61, which are higher than the present study (31).

The values of the weighted Kappa were significant for 53.3% of the nutrients, with slight to moderate agreement (0.17 to 0.46) in the present study and comparable to those from Martinez et al. (31), which ranged from 0.13 for monounsaturated fat to 0.40 for carbohydrate. Mascarenhas et al. (8) observed good correlation for all of the evaluated nutrients, with Kappa values between 0.47 for energy and 0.73 for iron, which are higher than those of the present study.

In conclusion, the FFQ presented an acceptable relative validity for lipids, saturated fatty acids, fiber, calcium, iron, riboflavin, niacin, vitamin C, and sodium. This instrument will be useful in future studies about dietary intake of adolescents in São Luís, Maranhão, and adequate to evaluate lipids and micronutrients.
